# Transforming Life Science Through Chromosome‐Level Genome Assemblies

**DOI:** 10.1111/gtc.70145

**Published:** 2026-08-21

**Authors:** Tetsuo Kon, Kosuke Kataoka, Yi‐Jyun Luo, Johannes Nicolaus Wibisana, Kouhei Toga, Narumi Uno, Yuki Monden, Mayuko Hamada

**Affiliations:** ^1^ Department of Neurosciences and Developmental Biology University of Vienna Vienna Austria; ^2^ Graduate School of Engineering Tokyo University of Agriculture and Technology Tokyo Japan; ^3^ Comprehensive Research Organization Waseda University Tokyo Japan; ^4^ Biodiversity Research Center Academia Sinica Taipei Taiwan; ^5^ Genomics and Regulatory Systems Unit Okinawa Institute of Science and Technology Graduate University Onna‐son Okinawa Japan; ^6^ Laboratory of BioDX, PtBio Co‐Creation Research Center, Genome Editing Innovation Center Hiroshima University Hiroshima Japan; ^7^ Laboratory of Genome Informatics, Graduate School of Integrated Sciences for Life Hiroshima University Hiroshima Japan; ^8^ Laboratory of Bioengineering School of Life Sciences, Tokyo University of Pharmacy and Life Sciences Tokyo Japan; ^9^ Graduate School of Environmental, Life, Natural Science, and Technology Okayama University Okayama Japan; ^10^ Ushimado Marine Institute, Okayama University Okayama Japan

**Keywords:** chromosome‐level genome assembly, genome evolution, Hi–C scaffolding, long‐read sequencing, non‐model animals

## Abstract

Advances in long‐read sequencing and Hi–C scaffolding have made chromosome‐level genome assembly increasingly accessible to individual laboratories, shifting genome research from large consortium‐led projects toward investigator‐driven studies across diverse taxa. This transition allows researchers to select organisms based on biological questions rather than the prior availability of genomic resources. In this review, we summarize the core experimental and computational steps for generating, evaluating, and annotating chromosome‐level assemblies, and examine how they have advanced research in non‐model organisms and genetically complex systems. Representative case studies illustrate four major contributions: resolving structural variation and lineage‐specific genome architecture, linking genome organization to phenotypic innovation and plasticity, reconstructing deep chromosome evolution and macrosynteny, and distinguishing homologous and homoeologous chromosomes in polyploid genomes. These examples show that chromosome‐level assemblies provide more than complete reference sequences. They establish a continuous genomic coordinate system through which genes, regulatory elements, transposable element insertions, sequence variants, and cellular states can be interpreted within broader chromosomal, population, and evolutionary contexts. We describe this integrative perspective as “glocal biology.” Future progress will require pangenomic, population‐scale, and haplotype‐resolved resources integrated with multi‐omics and functional analyses. Collectively, chromosome‐level genomics is reshaping life science by embedding molecular functions within chromosomal and evolutionary contexts.

## Graphical abstract

Chromosome‐level genome assemblies provide a framework for “glocal biology” in species of interest, linking local molecular features to chromosome architecture and evolutionary context. When integrated with pangenome analysis, comparative multi‐omics, genome language models, and experimental approaches, these assemblies enable hypothesis generation and testing, ultimately driving biological discovery across multiple scales.

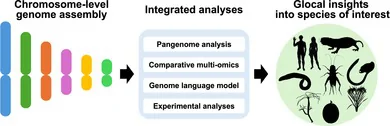

## 1 Transitioning From Consortium‐Based Genomics to Investigator‐Driven Chromosome‐Level Genomics

Early chromosome‐scale genome sequencing was exemplified by the Human Genome Project (HGP), which required extensive international collaboration, clone‐based sequencing, and physical mapping to reconstruct genome organization (Collins et al. 2003; International Human Genome Sequencing Consortium 2004; Lander et al. 2001). Because of the substantial infrastructure, cost, and coordination required, chromosome‐level assemblies were initially restricted to a small number of intensively studied model organisms. The subsequent introduction of short‐read sequencing greatly increased throughput and expanded the number of available draft genomes. However, short reads generally could not resolve long repetitive elements, segmental duplications, and structural variants, and therefore often produced fragmented assemblies (van Dijk et al. 2018; Mardis 2008; Pollard et al. 2018).

**FIGURE 1:**
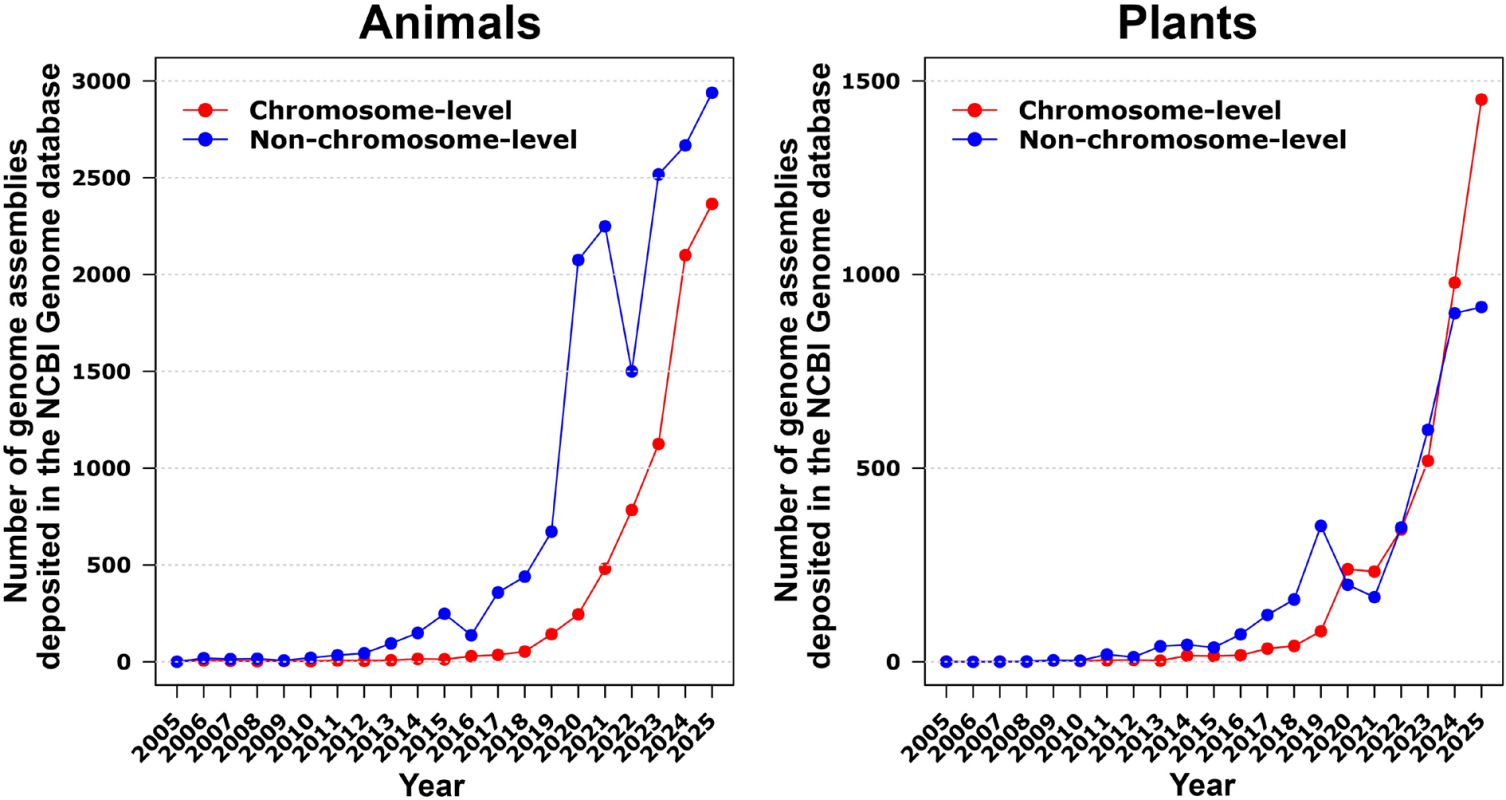
The rapid accumulation of chromosome‐level genome assemblies These plots show the annual number of genome assemblies deposited in the NCBI Genome database (2005–2025) for animals (left) and plants (right). Red indicates chromosome‐level assemblies, and blue indicates non‐chromosome‐level (contig‐level and scaffold‐level) assemblies. Note the exponential growth of chromosome‐level assemblies since around 2018.

A fundamental transition occurred with the development of long‐read sequencing and proximity ligation‐based scaffolding. Long reads can span repetitive and structurally complex regions, allowing the construction of highly contiguous sequences, whereas Hi–C provides long‐range linkage information that enables contigs to be ordered, oriented, and assigned to chromosomes (Burton et al. 2013; Kaplan and Dekker 2013). Their combination has made chromosome‐level genome assembly increasingly accessible to individual laboratories and small research groups (Ito et al. 2025; Kon‐Nanjo et al. 2023; Kon et al. 2025) (Figure 1, Table 1). Consequently, chromosome‐level genome assembly has shifted from a predominantly centralized, consortium‐driven model to an investigator‐driven approach applicable across the tree of life.

**TABLE 1:** Numbers of chromosome‐level genome assemblies.

Taxonomic group	Number of chromosome‐level genome assemblies^a^	Examples of species with chromosome‐level genome assemblies
Metazoa (animals)	7435	
Bilateria/Deuterostomia	3239	*Homo sapiens* (Nurk et al. 2022), *Protopterus annectens* (Schartl et al. 2024), *Ambystoma mexicanum* (Nowoshilow et al. 2018), *Garra rufa* (Kon et al. 2025), *Oikopleura dioica* (Bliznina et al. 2021), *Branchiostoma floridae* (Simakov et al. 2020)
Bilateria/Protostomia	3995	*Gryllus bimaculatus* (Kataoka, Sanno, et al. 2026), *Apis cerana* (Lee et al. 2025), *Octopus vulgaris* (Destanović et al. 2023), *Placopecten magellanicus* (Xie et al. 2023), *Alentia gelatinosa* (Adkins et al. 2023)
Cnidaria	116	*Hydra vulgaris* (Cazet et al. 2023; Kon‐Nanjo et al. 2025; Simakov et al. 2022), *Hydractinia symbiolongicarpus* (Kon‐Nanjo et al. 2023), *Nanomia septata* (Ahuja et al. 2026), *Nematostella vectensis* (Zimmermann et al. 2023), *Acropora palmata* (Locatelli et al. 2024)
Placozoa	0	Not available
Porifera	76	*Spongilla lacustris* (Leys et al. 2025), *Diacarnus erythraeanus* (Steindler et al. 2025)
Ctenophora	5	*Hormiphora californensis* (Schultz et al. 2021), *Bolinopsis microptera* (Schultz et al. 2023), *Mnemiopsis leidyi* (Kim et al. 2025)
Viridiplantae (green plants)	3987	
Spermatophyta	3695	*Triticum aestivum* (Walkowiak et al. 2020), *Glycine max* (Zhang, Shao, et al. 2024), *Solanum lycopersicum* (Li et al. 2023), *Citrullus lanatus* (Zhang, Zhao, et al. 2024), *Coffea arabica* (Salojärvi et al. 2024), *Gossypium hirsutum* (Hu et al. 2019)

^a^ The numbers represent the total number of chromosome‐level genome assemblies deposited in the NCBI Genome Database by the end of 2025. Subcategories shown are not exhaustive.

This shift allows researchers to select organisms on the basis of distinctive biological traits and evolutionary questions rather than the prior availability of genomic resources. More importantly, chromosome‐level assemblies provide a continuous genomic coordinate system in which local molecular features, such as genes, regulatory elements, transposable element insertions, and sequence variants, can be interpreted within broader chromosomal, regulatory, and evolutionary contexts. Together with advances in genetic manipulation and multi‐omics approaches, this makes it possible to investigate how local gene functions and cell‐type‐specific regulatory states are embedded within global chromosome architecture. We refer to this integrative framework as “glocal biology.” Here, the term does not denote a separate subdiscipline but describes an approach that connects multiple layers of biological information through chromosome‐resolved genome coordinates. In this sense, glocal biology complements comparative genomics, regulatory genomics, 3D genome biology, and evolutionary genomics by integrating their findings across cell types, populations, species, and evolutionary timescales. In this review, we first summarize the core workflow required to generate and evaluate chromosome‐level genome assemblies. We then highlight representative studies showing how chromosome‐level assemblies have connected local genomic findings with broader chromosome‐scale contexts in non‐model organisms across diverse taxa, before discussing their implications for genome evolution and experimental chromosome‐scale genomics.

## 2 Technological Foundations of Chromosome‐Level Genome Assembly

### 2.1 Core Steps of Modern Genome Assembly

**FIGURE 2:**
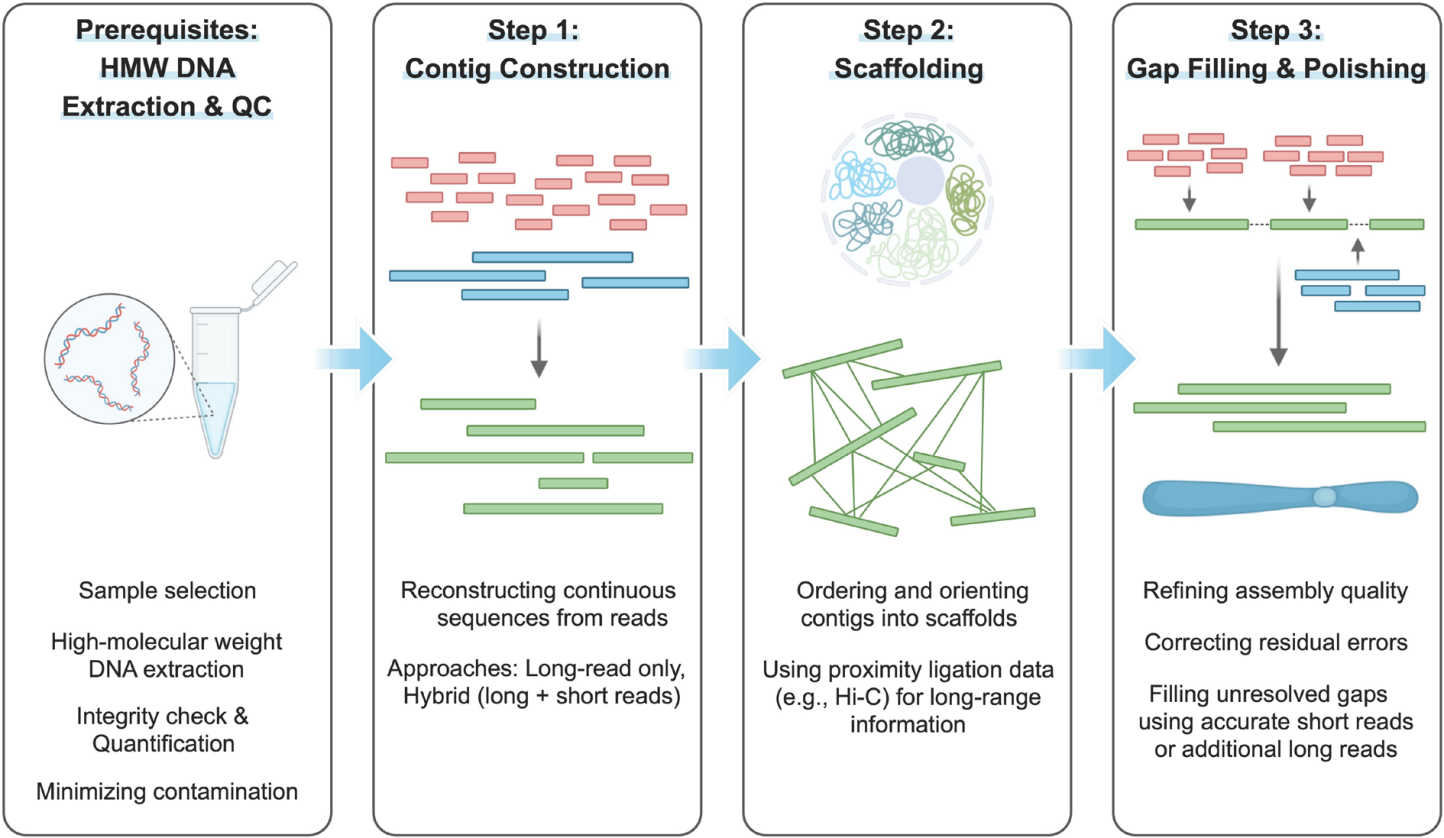
Conceptual workflow of modern chromosome‐level genome assembly Chromosome‐level genome assembly proceeds through a series of coordinated experimental and computational steps. The process begins with the extraction and quality control of high‐molecular‐weight (HMW) DNA, where sample integrity, quantification, and contamination minimization are critical for maximizing long‐read performance. In Step 1, contigs (green bars) are constructed by assembling sequencing reads into continuous sequences, typically using long‐read‐based or hybrid assembly strategies incorporating long reads (blue bars) and short reads (red bars). Step 2 employs proximity ligation‐derived information, such as Hi–C contact data, to order and orient contigs into chromosome‐level scaffolds by leveraging long‐range genomic interactions. Finally, Step 3 focuses on gap filling and polishing, where additional long reads or high‐accuracy short reads are used to correct residual errors and improve assembly completeness. Together, these stages illustrate how advances in sequencing and scaffolding technologies enable the reconstruction of genome assemblies that approximate native chromosomal organization. This figure was created in BioRender (https://BioRender.com/f8nu3xi).

Despite the diversity of sequencing platforms and analytical tools currently available, most modern genome assembly projects follow a common conceptual framework. At its core, chromosome‐level genome assembly can be understood as a three‐step process: contig construction, scaffolding, and gap filling or polishing (Figure 2). In practice, however, these steps are often iterative and may be interleaved, with polishing or gap filling occurring both during and after contig construction depending on the assembly strategy and data types used.

Before any computational assembly begins, high‐molecular‐weight (HMW) DNA extraction constitutes a foundational prerequisite for successful long‐read‐based genome assembly (Figure 2). Regardless of sequencing platform, DNA integrity directly constrains achievable read length and therefore the ability to resolve repetitive and structurally complex genomic regions. Equally important is minimizing contamination from symbionts, microbiota, or environmental DNA, as contaminant sequences can complicate assembly graphs and compromise downstream analyses. In practice, however, complete removal of symbiont‐derived DNA is not always feasible, particularly in organisms with intimate host–microbe associations, and such sequences are often identified and separated at the contig or scaffold level during downstream analyses. Routine quality control using electrophoresis systems such as TapeStation (Agilent Technologies), which assesses size distribution and fragmentation, and fluorometers such as Qubit (Thermo Fisher Scientific) and Quantus (Promega), which provide accurate double‐stranded DNA quantification, allows researchers to evaluate whether extracted DNA meets the requirements for long‐read sequencing. In practice, careful optimization of sample handling and extraction protocols often has a greater impact on downstream assembly outcomes than subsequent computational choices.

The first computational step, contig construction, aims to reconstruct continuous genomic sequences directly from sequencing reads (Figure 2). In contemporary workflows, this step is typically driven by long‐read sequencing technologies, which can span repetitive and structurally complex regions that previously fragmented assemblies. Contigs may be generated using long‐read‐only approaches or hybrid strategies that combine long reads with short reads. In diploid, hybrid, or polyploid genomes, haplotype‐aware assembly and strategies such as trio binning can separate homologous chromosome sequences, thereby reducing heterozygosity‐induced fragmentation and enabling analyses of allelic and subgenomic variation.

The second step, scaffolding, focuses on ordering and orienting contigs into larger structures that correspond to whole chromosomes (Figure 2). Proximity ligation‐based methods, most notably Hi–C, provide long‐range interaction information that reflects the three‐dimensional organization of chromosomes in the nucleus. This information enables contigs to be linked across megabase scales, forming chromosome‐level scaffolds. Reference‐guided scaffolding methods can also be useful when chromosome‐level assemblies of closely related species are available, particularly in organisms with relatively conserved karyotypes.

Finally, gap filling and polishing refine the assembly by correcting residual errors and filling unresolved gaps (Figure 2). This step typically leverages the high accuracy of short reads or additional long‐read data to improve base‐level quality and overall completeness. Together, these three steps form the technological backbone of modern chromosome‐level genome assembly.

### 2.2 Long‐Read Sequencing as the Foundation of Modern Genome Assembly

Long‐read sequencing has become the backbone of modern genome assembly. By producing reads that span repetitive elements and structurally complex regions, long‐read technologies allow complex regions to be resolved by physically linking sequences that short reads cannot bridge. This capability is particularly critical for repeat‐rich or large genomes, in which transposable elements, segmental duplications, and long introns routinely exceed the length of short reads.

Two major long‐read technologies are currently used for genome assembly: single‐molecule real‐time sequencing, implemented by Pacific Biosciences (PacBio), and nanopore sequencing (Goodwin et al. 2016). PacBio HiFi sequencing generates long reads with high per‐base accuracy by repeatedly sequencing circularized DNA molecules. Nanopore sequencing detects changes in ionic current as individual DNA molecules pass through a nanoscale pore and can generate ultra‐long reads extending to hundreds of kilobases or more. Nanopore sequencing can often be performed within individual laboratories, providing flexibility for pilot experiments and protocol optimization, whereas PacBio sequencing is more commonly conducted at centralized facilities and is particularly well suited for accurate contig construction and haplotype‐aware assembly.

Long‐read assemblers convert these data into contiguous genome sequences. Hifiasm is widely used for accurate and haplotype‐resolved assembly of PacBio HiFi data (Cheng et al. 2021), whereas Flye supports both PacBio and nanopore reads across diverse genome architectures (Kolmogorov et al. 2019). Canu remains an established option, particularly for noisy long‐read datasets (Koren et al. 2017). Additional assemblers, including wtdbg2 (Ruan and Li 2020), NextDenovo (Hu et al. 2024), Verkko (Rautiainen et al. 2023), and NOVOloci (Dierckxsens et al. 2025), provide alternative strategies suited to different read types, genome structures, and levels of heterozygosity. In parallel, hybrid assembly approaches that combine long reads with high‐accuracy short reads continue to be used. Assemblers such as MaSuRCA (Zimin et al. 2013), the hybrid modes of SPAdes (Prjibelski et al. 2020), and graph‐based methods such as WENGAN (Di Genova et al. 2021) integrate complementary data types to improve assembly contiguity and accuracy. The optimal platform and assembly strategy ultimately depend on genome architecture, DNA quality and quantity, and the required balance among read length, accuracy, contiguity, and haplotype resolution.

### 2.3 Hi–C Scaffolding: From Contigs to Chromosomes

Hi–C‐based scaffolding enables chromosome‐level genome assembly by converting three‐dimensional chromatin proximity information into one‐dimensional genomic order (Figure 2). Genomic regions located near one another on the same chromosome generally interact more frequently than regions on different chromosomes or at distant positions, allowing Hi–C contact frequencies to be used to infer contig adjacency, orientation, and chromosomal assignment. In contact maps, correctly assembled chromosomes typically form dense interaction blocks, whereas misassemblies produce discontinuities or discordant interaction patterns. Hi–C, therefore, serves not only as a scaffolding method but also as an important quality‐control approach for detecting and correcting assembly errors.

Several proximity ligation methods and computational tools are available for this purpose. Classical Hi–C relies on restriction enzyme digestion of crosslinked chromatin, whereas commercial implementations such as Arima Genomics optimize enzyme combinations for more uniform coverage. Alternative approaches include Chicago libraries, which use in vitro reconstituted chromatin, and Omni‐C, which employs endonuclease‐based fragmentation independent of restriction enzyme recognition sites. Computational pipelines commonly use Juicer to generate normalized contact maps (Durand, Shamim, et al. 2016), followed by scaffolders such as 3D‐DNA (Dudchenko et al. 2017) or YaHS (Zhou et al. 2023) to construct chromosome‐level assemblies. When Hi–C data are unavailable, reference‐guided tools such as RagTag can provide an alternative scaffolding strategy (Alonge et al. 2022). Finally, interactive visualization with Juicebox remains important for manual curation and the resolution of complex assembly structures (Durand, Robinson, et al. 2016).

### 2.4 Assembly Evaluation and Genome Annotation

Finally, chromosome‐level genome assemblies must be evaluated for both structural continuity and biological completeness. Assembly quality is commonly assessed using scaffold‐ and contig‐level statistics together with gene space completeness metrics provided by tools such as BUSCO (Tegenfeldt et al. 2025), compleasm (Huang and Li 2023), and OMArk (Nevers et al. 2025), which benchmark assemblies against conserved ortholog sets and expected gene architectures. Base‐level accuracy and completeness can be further examined using k‐mer‐based approaches such as Merqury (Rhie et al. 2020), which compare sequencing reads and assembled genomes without requiring reference sequences. Optical mapping can provide independent, ultra‐long‐range validation of scaffold structure, helping to confirm chromosome‐scale organization and identify potential misassemblies. In parallel, contamination screening helps identify sequences derived from symbionts, microbiota, or environmental sources that may otherwise confound downstream analyses. Dedicated tools such as FCS‐GX (Astashyn et al. 2024) and BlobToolKit (Challis et al. 2020) support taxonomic assignment and coverage‐based visualization, facilitating the detection and removal of contaminant sequences in complex assemblies. Integrated QC pipelines such as GenomeQC (Manchanda et al. 2020) can also be useful for streamlining evaluation.

Beyond assembly evaluation, accurate gene prediction and annotation are essential for downstream biological analyses but remain challenging in non‐model organisms, particularly when transcriptomic resources, curated proteins, or closely related reference genomes are limited. Recent pipelines such as NCBI EGAPx (Goldfarb et al. 2025) and BRAKER (Gabriel et al. 2024) integrate protein homology, RNA‐seq evidence, and ab initio prediction. For species with well‐annotated reference genomes, annotation transfer tools such as Liftoff (Shumate and Salzberg 2021) and LiftOn (Chao et al. 2025), as well as comparative annotation frameworks such as TOGA (Kirilenko et al. 2023), provide complementary approaches for projecting gene models onto newly assembled genomes. However, organisms with unusual gene architectures, including trans‐splicing, very short introns, or compact intergenic regions, may still require species‐specific optimization. Annotation strategies should also reflect the intended downstream applications. Cap analysis of gene expression (CAGE) data can accurately identify transcription start sites, thereby improving the annotation of 5′ transcript ends and enabling the inference of alternative promoter usage, whereas long‐read RNA sequencing can resolve full‐length transcript isoforms and improve the annotation of 3′ untranslated regions. Such improvements are especially important for single‐cell RNA‐seq because incomplete gene models can reduce read assignment and obscure cell‐type‐specific expression patterns. Functional annotation is also challenging for lineage‐specific genes because experimentally characterized homologs are often absent from existing databases.

## 3 Case Studies: Conceptual Advances Enabled by Chromosome‐Level Genome Assemblies

**FIGURE 3:**
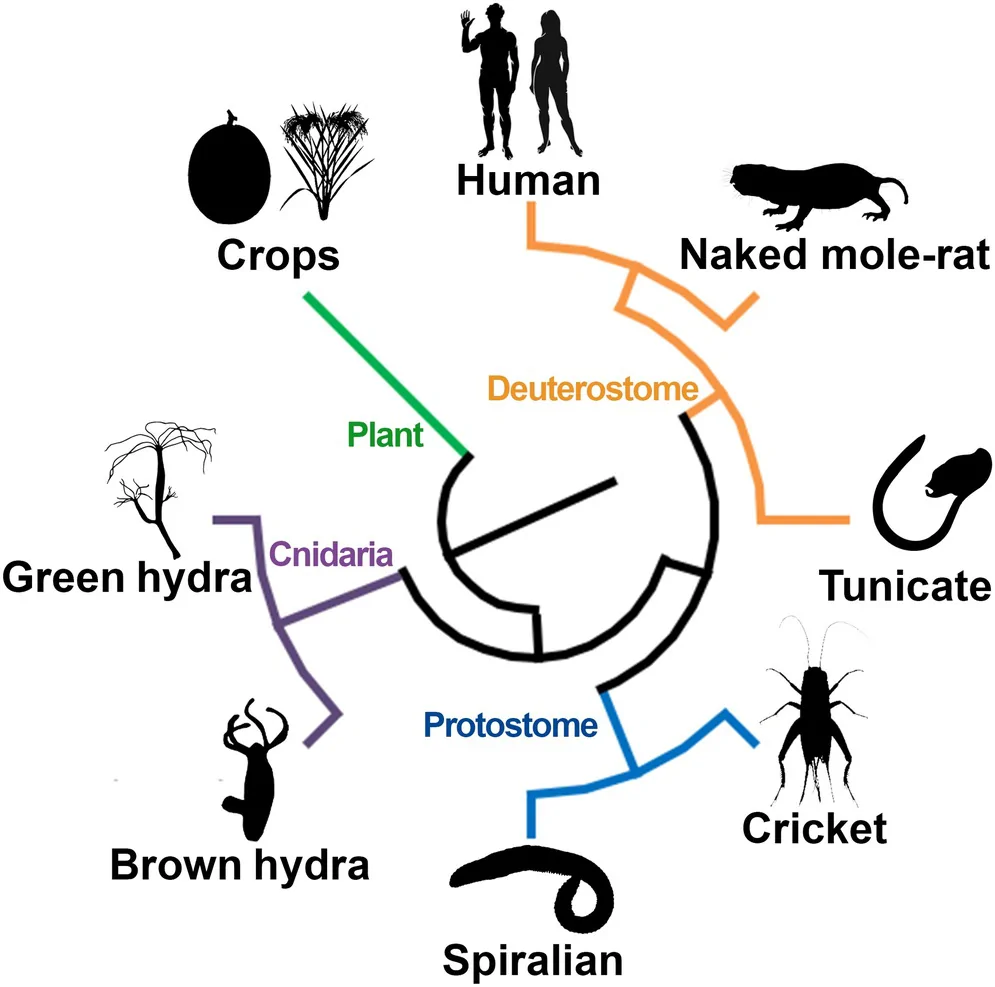
Cladogram of representative taxa in which chromosome‐level genome assemblies have advanced molecular and evolutionary analyses Animal silhouettes were obtained from the PhyloPic database (https://www.phylopic.org/). The cladogram was constructed using MEGA12 software (Kumar et al. 2024).

The following case studies cover representative taxa across the tree of life (Figure 3) and are organized into four conceptual categories based on the biological questions addressed by chromosome‐level genome assemblies. First, chromosome‐resolved genomes reveal structural variation and lineage‐specific genome architecture that are obscured in fragmented genome assemblies. Second, they provide a framework for linking genome organization to phenotypic innovation and developmental plasticity. Third, comparisons of chromosome‐level assemblies enable the reconstruction of deep chromosome evolution and conserved macrosynteny. Finally, chromosome‐level assemblies make it possible to analyze genetically complex systems, including highly heterozygous and polyploid genomes. Together, these examples illustrate how chromosome‐level genome assemblies provide biological insights that cannot be obtained from gene inventories or fragmented draft genomes alone.

### 3.1 Resolving Structural Variation and Lineage‐Specific Genome Architecture

#### 3.1.1 Genome Scrambling and Hidden Lineage Diversity in Larvaceans

Larvaceans (appendicularians) are tunicates and, therefore, represent an important comparative reference lineage for understanding chordate genome evolution before vertebrate‐specific whole‐genome duplications (Dehal and Boore 2005; Donoghue and Purnell 2005). Among them, *Oikopleura dioica* is particularly informative because it combines an extremely compact genome with extensive lineage‐specific gene loss, gene duplication, and chromosome rearrangement (Albalat and Cañestro 2016; Ferrández‐Roldán et al. 2019; Martí‐Solans et al. 2021). This raises the question of how morphologically similar lineages can retain a conserved chordate body plan despite profound differences in genome content and chromosome organization.

The first *O. dioica* reference genome revealed an unusually compact genome of approximately 60 Mb containing more than 15,000 genes, widespread operons, and highly reduced introns (Denoeud et al. 2010; Ganot et al. 2004). Subsequent long‐read and chromosome‐level assemblies from geographically distinct populations revealed that *O. dioica* is not a single genetically uniform species but a complex of deeply divergent lineages (Bliznina et al. 2021; Masunaga et al. 2022; Plessy et al. 2024; Wang et al. 2020). Whole‐genome comparisons identified extensive differences in genome content and chromosome organization despite limited morphological differentiation, supporting the existence of multiple cryptic lineages and raising fundamental questions concerning species boundaries and phenotypic conservation (Masunaga et al. 2022; Plessy et al. 2024).

Accurate interpretation of these differences depends on assemblies that preserve native chromosome structure. *O. dioica* presents several technical challenges, including low DNA yield, uneven heterozygosity between chromosome arms, frequent inversions, and long homopolymeric sequences in the mitochondrial genome (Bliznina et al. 2021; Dierckxsens et al. 2024; Plessy et al. 2024). Fragmented assemblies and reference‐guided scaffolding may obscure genuine inversions and other lineage‐specific rearrangements. NOVOloci has recently been used with Oxford Nanopore data, even from earlier R9 chemistry, to generate near‐complete or complete chromosome arms as single contigs, reducing reliance on reference‐guided scaffolding and enabling more direct comparisons of chromosome structure among lineages (Dierckxsens et al. 2025).

Chromosome‐level assemblies have therefore transformed *O. dioica* research from the analysis of a single compact reference genome into the comparative study of structurally divergent genomes. However, currently available assemblies do not yet capture the full geographic diversity of the species complex, particularly in the Southern Hemisphere. Existing resources, including ANISEED, TUNOME, and lineage‐specific genome databases, remain distributed across separate platforms (Brozovic et al. 2018; Shito et al. 2025). The development of integrated pangenomic and multi‐omics resources will be essential for determining how chromosome‐scale rearrangements reshape local gene content and regulation, and how these changes are integrated to preserve a conserved body plan while permitting lineage‐specific adaptation.

#### 3.1.2 Transposable Element‐Driven Genome Expansion and Stem‐Cell Genome Diversification in *Hydra*

The genomes of brown hydra, including *Hydra vulgaris*, are unusually large for cnidarians and are strongly enriched in transposable elements. The first *H. vulgaris* genome was generated through a large international consortium using clone‐based Sanger sequencing and consisted of more than 20,000 sequence fragments (Chapman et al. 2010). Although this fragmented assembly revealed substantial expansion of chicken repeat 1 retrotransposons, it could not fully resolve the chromosomal distribution, copy‐number structure, or lineage‐specific activity of transposable elements.

Subsequent chromosome‐level and telomere‐to‐telomere assemblies, together with single‐cell and epigenomic datasets, have provided a chromosome‐resolved view of transposable element evolution in *Hydra* (Cazet et al. 2023; Kon‐Nanjo et al. 2025; Siebert et al. 2019; Simakov et al. 2022). These assemblies revealed that genome expansion is not attributable to a single repeat family but involves multiple anciently active transposable element families, including DNA transposons such as hAT‐Charlie and TcMar‐Tc1. They further showed that the three continuously self‐renewing stem‐cell lineages of *Hydra* have accumulated distinct transposable element insertions during prolonged asexual propagation, and suggested that some of these insertions may influence lineage‐specific gene expression (Kon‐Nanjo et al. 2025).

These findings depended on highly contiguous reference genomes because transposable elements and their insertion sites cannot be reliably assigned to genomic context in fragmented assemblies. Chromosome‐level and telomere‐to‐telomere assemblies made it possible to distinguish genuine lineage‐specific insertions from assembly fragmentation, compare repeat distributions across entire chromosomes, and investigate how transposable element activity contributes to both genome expansion and somatic genome diversification. Together, the *Oikopleura* and *Hydra* examples illustrate how chromosome‐level assemblies connect large‐scale genome restructuring, including inversions and repeat expansion, with local changes in gene content, transposable element insertions, and gene regulation, thereby revealing how lineage‐specific genome architecture may shape biological variation.

### 3.2 Linking Genome Organization to Phenotypic Innovation and Plasticity

#### 3.2.1 Genome Architecture and the Evolution of Photosymbiosis in Green Hydra

*Hydra* can serve not only as a model for the transposon‐driven evolution of genome architecture described above, but also as a model for understanding how interspecies interactions shape phenotypic and genomic evolution. *Hydra viridissima*, commonly known as green hydra, forms a stable intracellular photosymbiosis with the green alga *Chlorella*. The symbiont resides within endodermal epithelial cells and supplies photosynthetically derived carbohydrates to the host, whereas the host supplies nitrogen‐containing metabolites to the alga (Davy et al. 2012). Unlike brown hydra, which possess genomes of approximately 1 Gb enriched in transposable elements, the green *Hydra* genome is approximately 300 Mb and contains a substantially smaller proportion of retrotransposons (Hamada et al. 2020; Wong et al. 2019). This contrast provides a comparative system for studying how genome architecture, transposable element expansion, and symbiotic innovation evolved within a single genus.

Short‐read genome assemblies of *H. viridissima* strain A99 and its symbiotic *Chlorella* enabled initial analyses of gene content and metabolic interdependence (Hamada et al. 2018, 2020). Photosynthetically produced maltose is associated with increased expression of host genes involved in nutrient assimilation and transport, including glutamine synthetase and phosphate transporters. Conversely, the symbiotic alga lacks several genes required for autonomous inorganic nitrogen assimilation and instead depends on host‐derived amino acids.

Chromosome‐level genomes are now becoming available for green *Hydra*, including an assembly from a US population (NCBI Genome accession: GCF_964215635.1), with additional resources for strain A99 under development. These assemblies will allow direct chromosome‐scale comparisons between green and brown hydra, including the reconstruction of lineage‐specific transposable element expansions, structural rearrangements, and regulatory landscapes. They will also provide more accurate gene models for single‐cell and spatial transcriptomic analyses, which are particularly important for distinguishing host and symbiont transcription within individual cell types. The green *Hydra* example therefore extends chromosome‐level genomics from the description of genome size differences to mechanistic questions concerning photosymbiosis, species diversification, and phenotypic evolution.

#### 3.2.2 Structural and Intraspecific Genomic Variation Underlying Naked Mole‐Rat Phenotypes

The naked mole‐rat, *Heterocephalus glaber* , exhibits a distinctive combination of exceptional longevity, cancer resistance, hypoxia tolerance, reduced thermoregulation, and eusociality. These traits make it a valuable comparative system for identifying genomic changes associated with physiological innovations that are not readily modeled in conventional laboratory rodents (Buffenstein 2008; Buffenstein and Pinto 2009).

Initial genomic studies identified several species‐specific gene changes potentially associated with these phenotypes. These include substitutions in the telomere‐associated protein TERF1, alterations in the thermogenic gene *UCP1*, natural loss‐of‐function mutations in the necroptosis genes *RIPK3* and *MLKL*, and naked mole‐rat‐specific substitutions in cGAS that enhance homologous recombination repair (Chen et al. 2025; Oka et al. 2022). Such findings demonstrate the value of gene‐level comparative analyses, but they do not reveal how larger‐scale genomic changes, including structural variants, tandem duplications, and population‐specific variation, contribute to naked mole‐rat phenotypes.

Independent sequencing of naked mole‐rats maintained in Japan revealed substantial intraspecific variation and highlighted limitations of relying on a single reference genome (Toga et al. 2026). Comparative analyses identified 77 transcripts with clear variation, including genes encoding amine ligand‐binding receptors such as *Htr2b* and *Gpr143*. Population divergence among naked mole‐rats had previously been detected using mitochondrial markers, with some geographically separated groups approaching levels of divergence observed between recognized species of bathyergid rodents (Faulkes et al. 1997; Ingram et al. 2015). Chromosome‐level assemblies from multiple populations will therefore be necessary to determine whether structural and allelic variants contribute to differences in physiology, behavior, or disease resistance.

More recent long‐read chromosome‐level assemblies have resolved genomic regions that were poorly represented in short‐read references (Sokolowski et al. 2024). Thousands of repetitive elements were newly resolved, and the olfactory receptor gene repertoire was found to span approximately 17 Mb rather than the 13 Mb represented in the previous reference. The assembly also identified tandem duplications involving biologically relevant genes, including *TINF2*, which is associated with telomere biology and hypoxia tolerance, and *TCP1*, which functions in protein folding and has been implicated in tumor progression. These structural features could not be reliably identified in fragmented short‐read assemblies.

The naked mole‐rat example therefore demonstrates that chromosome‐level genomics extends phenotype‐oriented comparative studies beyond individual coding mutations. By resolving gene‐family expansions, tandem duplications, repetitive regions, and population‐specific structural variation, chromosome‐level assemblies provide a more complete framework for testing how exceptional physiological traits emerge and vary within a species.

#### 3.2.3 Chromosomal and Epigenetic Regulation of Developmental Fate Plasticity in Crickets

Crickets belong to Polyneoptera, an insect lineage that is phylogenetically distant from the holometabolous species that dominate classical insect genetics. They exhibit diverse environmentally responsive traits, including changes in wing morphology, reproduction, metabolism, stress tolerance, and developmental timing (Bonfoey et al. 2023; Harrison 1979; Kasumovic et al. 2016). These traits provide an opportunity to investigate how a single genome produces alternative phenotypes through context‐dependent regulation.

The band‐legged ground cricket *Dianemobius nigrofasciatus* is a particularly informative system because maternal photoperiod determines whether genetically similar embryos enter diapause or proceed through direct development (Masaki and Sugahara 1992; Shiga and Numata 1997). Diapause‐destined and nondiapause embryos are initially morphologically indistinguishable but later diverge when nondiapause embryos initiate germ‐band formation and diapause‐destined embryos arrest at the cellular blastoderm stage (Tanigawa et al. 2009). The central question is therefore not which genes are present, but how alternative regulatory states are organized across the same genome before visible phenotypic divergence.

A highly contiguous genome assembly has enabled the gene regulatory landscape to be mapped and interpreted across continuous genomic regions. Whereas earlier studies relied primarily on transcriptome comparisons, integration of the chromosome‐level assembly with ATAC‐seq has revealed coordinated changes in chromatin accessibility at loci associated with metabolism, cell‐cycle regulation, and neural development (Kataoka, Shimizu, et al. 2026). Importantly, these chromatin‐state differences arise before strong transcriptional divergence becomes apparent.

This finding illustrates a limitation of purely gene‐centric explanations of developmental plasticity. Diapause and nondiapause embryos share the same genomic sequence, yet local regulatory elements are activated or repressed in coordinated patterns across contiguous genomic regions. Chromosome‐level assemblies are required to connect these regulatory elements to neighboring genes, repetitive sequences, and broader chromosomal context, thereby revealing how genome organization supports large‐scale and reversible changes in developmental fate.

Chromosome‐level references also improve functional testing. CRISPR/Cas9‐based editing has become increasingly feasible in crickets, and a continuous reference genome improves guide RNA design, target specificity, and genome‐wide assessment of potential off‐target sites (Matsuoka et al. 2025; Shimizu and Kataoka 2026; Shirai et al. 2022). Thus, the cricket case study illustrates how chromosome‐level assemblies connect environmental cues and local regulatory changes with their broader genomic context and organismal developmental outcomes in a non‐model insect.

Together, the green hydra, naked mole‐rat, and cricket case studies illustrate how chromosome‐resolved genomes connect local molecular changes and regulatory states to organism‐level phenotypes.

### 3.3 Reconstructing Deep Chromosome Evolution and Macrosynteny

Chromosome‐level genome assemblies have revealed that chromosome architecture can remain conserved across hundreds of millions of years, even when gene sequences and organismal morphology have diverged extensively. Orthologous genes retained on the same chromosomes define conserved macrosyntenic blocks, and comparisons across metazoans have enabled the reconstruction of 24 ancestral linkage groups in the last common bilaterian ancestor (Simakov et al. 2013, 2020, 2022). These ancestral linkage groups provide a framework for identifying chromosome fusions, fissions, and fusion‐with‐mixing events across deep evolutionary time.

Spiralians are particularly informative for such analyses because they include numerous animal phyla with contrasting chromosome architectures. Chromosome‐level assemblies from mollusks, annelids, nemerteans, bryozoans, and brachiopods have shown that many spiralian lineages retain substantial components of the ancestral bilaterian karyotype (Liao et al. 2023; Martín‐Zamora, Davies, et al. 2023). For example, the scallop *Pecten maximus* , the nemertean *Lineus longissimus* , and the annelid *Owenia fusiformis* retain most ancestral linkage groups as separate chromosomes (Kenny et al. 2020; Kwiatkowski et al. 2021; Martín‐Zamora, Liang, et al. 2023). Four fusion‐with‐mixing events shared across lophotrochozoans reduced the inferred ancestral chromosome number from 24 to 20 (Simakov et al. 2022).

Comparative chromosome analyses have also distinguished distinct modes of chromosome evolution, corresponding to low‐rearrangement lineages and high‐rearrangement lineages. In low‐rearrangement lineages, ancestral chromosomes are primarily combined through progressive fusion. In high‐rearrangement lineages, repeated fusion and fission events dismantle ancestral architecture (Lewin et al. 2024; Lewin, Liao, Luo 2025). The incidence of chromosome fission is a key feature distinguishing these two patterns of chromosome evolution.

Clitellate annelids represent an extreme example of genome restructuring. Whereas many marine annelids retain an ancestral complement of approximately 20 chromosomes, clitellate genomes show extensive dispersal of genes from all 24 ancestral bilaterian linkage groups across their chromosomes (Lewin et al. 2024). This pattern is consistent with a rapid episode of chromosome rearrangement rather than the gradual accumulation of isolated events. Losses of genes involved in genome stability and cell division, transposable element expansion, putative neocentromere formation, and whole‐genome duplication may all have contributed to this architectural disruption (Jin et al. 2020; Vargas‐Chávez, Benítez‐Álvarez, et al. 2025; Vargas‐Chávez, McLysaght, et al. 2025).

Extensive restructuring has also been identified in Bryozoa, a phylum for which chromosome‐scale genome organization was previously unknown. Analysis of five chromosome‐level genomes revealed that ancestral bilaterian linkage groups were extensively fused and rearranged before the diversification of living bryozoans, reducing the ancestral complement to approximately six linkage groups (Lewin, Liao, Chen, et al. 2025). Subsequent lineage‐specific fissions and rearrangements occurred in both gymnolaemate and phylactolaemate bryozoans, demonstrating that chromosome fission has played a larger role in bilaterian evolution than previously recognized.

Chromosome rearrangements can also serve as phylogenetic characters. Fusion‐with‐mixing events involve the interspersion of genes from two ancestral chromosomes and are unlikely to arise independently in the same configuration. Such rare genomic changes have helped resolve the position of ctenophores at the base of the animal tree and branching relationships among teleost fishes (Parey et al. 2023; Schultz et al. 2023). Within Spiralia, shared derived chromosome fusions between bryozoans, brachiopods, and phoronids support the monophyly of Lophophorata and place phoronids and bryozoans as sister groups (Lewin, Liao, Chen, et al. 2025; Lewin, Sakagami, Kao, et al. 2025).

These studies demonstrate that chromosome‐level assemblies contribute more than improved gene annotation. They reveal conserved ancestral chromosome units, identify irreversible structural changes, and provide phylogenetic information that complements sequence‐based analyses. As chromosome‐level assemblies accumulate across the tree of life, integration of macrosynteny, regulatory genomics, and epigenetic data will help explain why chromosome architecture remains stable in some lineages but undergoes rapid reorganization in others.

### 3.4 Enabling Genomics in Complex and Polyploid Genomes

Polyploid genomes contain more than two sets of chromosomes and are common among crop species, including cotton (4×), potato (4×), bread wheat (6×), sweetpotato (6×), and strawberry (8×). Their analysis is challenging because homologous and homoeologous chromosomes contain highly similar sequences, while allelic dosage and subgenome‐specific variation must be distinguished. Fragmented assemblies can collapse related chromosome copies, obscure structural variation, and prevent accurate assignment of genes and markers to individual subgenomes.

Historically, genomic analyses of polyploid crops often relied on diploid relatives. For example, the diploid *Fragaria vesca* genome was initially used as a reference for octoploid cultivated strawberry, and diploid *Ipomoea trifida* served as a reference for hexaploid sweetpotato (Hirakawa et al. 2015; Li et al. 2019; Shulaev et al. 2011; Wu et al. 2018). However, diploid relatives do not necessarily contain the genes, structural variants, or allelic combinations underlying important traits in cultivated polyploids.

Long‐read sequencing and Hi–C scaffolding have enabled direct chromosome‐level reconstruction of polyploid crop genomes. A phased chromosome‐level assembly of hexaploid sweetpotato, for example, resolved the architecture of individual chromosome copies and provides a substantially improved reference for breeding and genetic analysis (Wu et al. 2025). Similar advances in other polyploid crops have enabled subgenome assignment, detection of structural variation, and comparative analysis of chromosome evolution.

Chromosome‐level references are also essential for dosage‐aware genetic analysis. In polyploids, a biallelic locus can occur in multiple dosage states rather than as a single heterozygous genotype. Polyploid‐specific methods therefore estimate allele dosage from sequencing depth and incorporate this information into linkage mapping and genome‐wide association studies. Such approaches have been used to identify loci associated with disease resistance in hexaploid sweetpotato and to develop markers for breeding (Obata et al. 2022; Yamamoto et al. 2020). Tools such as MAPpoly additionally model the large number of possible gametic combinations and recombination patterns in polyploid populations (Mollinari et al. 2020).

The principal contribution of chromosome‐level assemblies in polyploid genomics is the ability to distinguish homologous and homoeologous chromosome copies, assign variants to the correct genomic background, and connect genotype dosage to phenotype. These capabilities transform polyploid genomes from incompletely resolved collections of related sequences into chromosome‐resolved resources that can support association mapping, marker‐assisted selection, and genomic breeding.

## 4 Future Perspectives: Synthesizing Lessons From Chromosome‐Level Genomics

**FIGURE 4:**
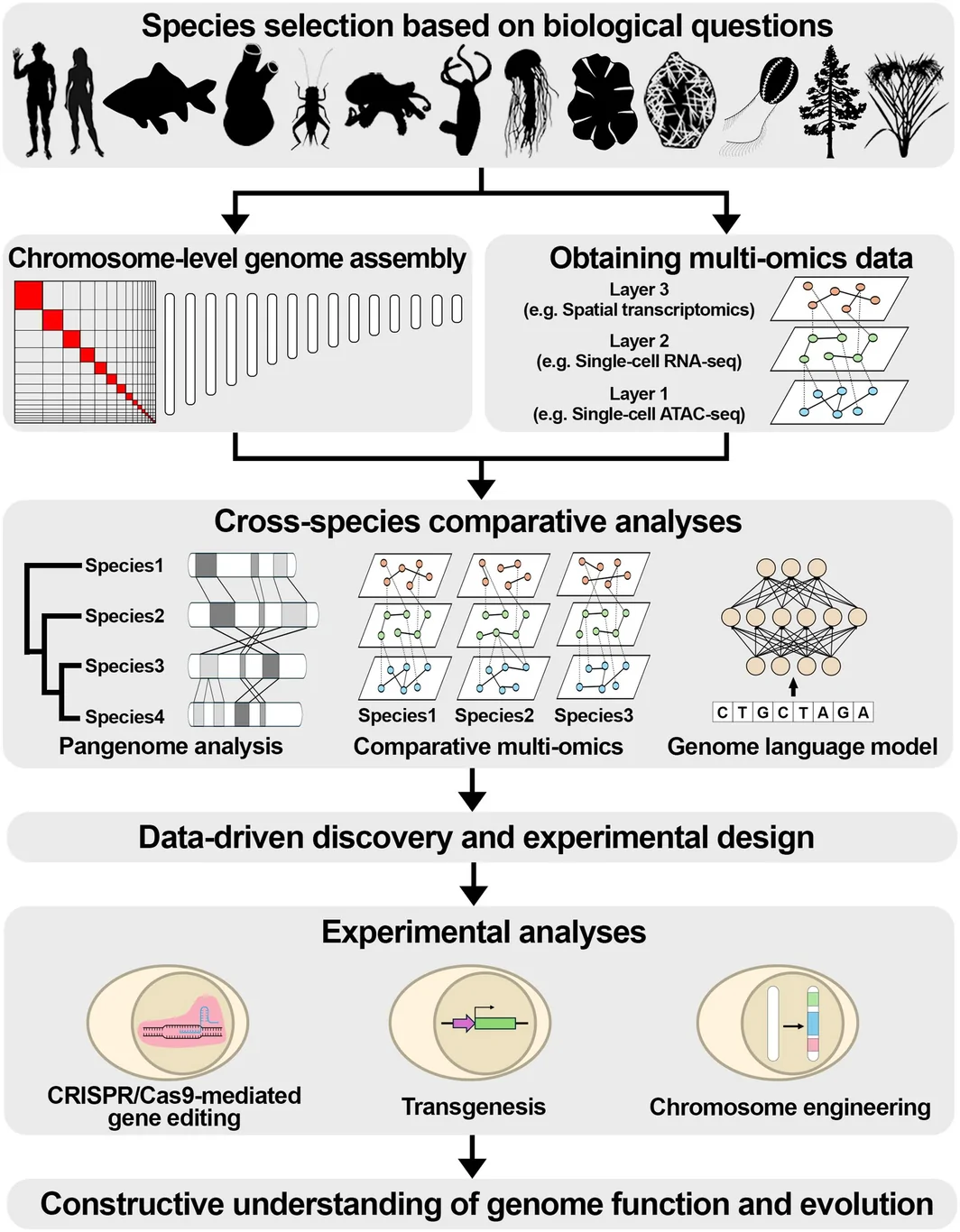
From chromosome‐level genome assembly to experimental chromosome‐scale genomics The integration of chromosome‐level genome assemblies with multi‐omics data and pangenome analyses, together with genome language models, facilitates data‐driven discovery and experimental design. Subsequent experimental validations, including CRISPR/Cas9‐mediated gene editing, transgenesis, and chromosome engineering, lead to a constructive understanding of genome function and evolution. The animal silhouettes were obtained from the PhyloPic database (https://www.phylopic.org/).

Taken together, the case studies reviewed here highlight several general lessons. First, chromosome‐level assemblies reveal structural features that cannot be reliably resolved or interpreted from fragmented assemblies, including large inversions, tandem duplications, chromosome‐wide transposable element landscapes, ancestral linkage groups, and relationships among homologous or homoeologous chromosomes. Second, genes, regulatory elements, sequence variants, and copy‐number differences can be interpreted more accurately when their positions and organization within chromosomes are known. Third, chromosome‐level assemblies become most informative when they are integrated with comparative, population genomic, epigenomic, and functional data. Finally, these integrated analyses generate hypotheses concerning genome structure, regulation, and evolution that can increasingly be tested experimentally. These lessons point toward four related future directions: moving from individual reference genomes to comparative chromosome resources, connecting local molecular findings with global chromosome organization, extending chromosome‐scale comparisons to evolutionary inference, and experimentally testing chromosome‐scale hypotheses (Figure 4).

### 4.1 Beyond a Single Reference Genome: Capturing Genomic Diversity Within and Among Species

The increasing accessibility of long‐read sequencing and Hi–C scaffolding has enabled individual laboratories to generate chromosome‐level assemblies for species selected on the basis of biological questions rather than the prior availability of genomic resources. The next step is to move beyond the generation of a single high‐quality reference for each species and to develop comparative genomic resources that represent variation among individuals, populations, lineages, and related species.

The case studies discussed in this review illustrate why this transition is necessary. Comparisons among geographically distinct *Oikopleura* lineages revealed extensive differences in genome content and chromosome organization despite limited morphological divergence. Studies of naked mole‐rats showed that a single reference genome cannot fully represent population‐specific sequence and structural variation. Comparisons between green and brown *Hydra* provide a framework for reconstructing lineage‐specific transposable element expansion, genome‐size evolution, and species diversification. In polyploid crops, multiple homologous and homoeologous chromosome copies must be distinguished to assign sequence variants and allele dosage to the correct genomic background.

Pangenomic, population‐scale, and haplotype‐resolved approaches will therefore become increasingly important. In highly variable, heterozygous, or polyploid genomes, a single linear reference may obscure structural variants, allelic differences, and subgenome‐specific features. Generating chromosome‐level assemblies for more individuals, populations, lineages, and related species will make it possible to distinguish conserved genome organization from individual, population, or lineage‐specific variation. The future value of chromosome‐level genomics will thus depend not only on the completeness of individual assemblies but also on their integration into comparative genomic resources that capture diversity within and among species.

### 4.2 From Local Findings to Glocal Biology

Chromosome‐level assemblies provide a common coordinate system through which local molecular observations can be interpreted within broader genomic contexts. The case studies reviewed here illustrate this principle at several levels. Transposable element insertions in Hydra can be related to chromosome‐wide repeat distributions and lineage‐specific gene regulation. Chromatin accessibility changes in crickets can be connected to neighboring genes, regulatory regions, and developmental programs. Structural variants and tandem duplications in naked mole‐rats can be evaluated within population‐specific chromosome architectures. In polyploid crops, allele dosage and sequence variation can be assigned to the appropriate homologous or homoeologous chromosome copies.

These examples define the practical basis of glocal biology. The term does not describe a separate field, but an integrative approach in which genes, regulatory elements, sequence variants, and cellular states are analyzed within chromosome‐resolved structural and evolutionary frameworks. Chromosome‐level assemblies make this integration possible by providing continuous genomic coordinates shared across gene annotation, epigenomic profiling, population variation, comparative genomics, and functional experiments.

Future studies should therefore combine chromosome‐level assemblies with single‐cell and spatial transcriptomics, chromatin accessibility and 3D genome data, population genomics, and phenotypic analyses. Such integration will allow cell‐type‐specific regulatory states and local molecular changes to be connected with organism‐level phenotypes and evolutionary differences. A central question will be not only where genes and regulatory elements are located, but also how their chromosomal context constrains or enables their regulation, variation, and evolutionary change. Addressing this question will help distinguish genomic features that merely record chromosome history from those that actively contribute to phenotypic innovation and plasticity.

### 4.3 From Chromosome‐Scale Comparisons to Evolutionary Inference

Chromosome‐level comparisons provide information about evolutionary history that cannot be recovered reliably from individual genes or fragmented genome sequences alone. The *Oikopleura*, *Hydra*, and spiralian case studies show that chromosome architecture may either remain deeply conserved or undergo extensive lineage‐specific reorganization. Comparisons of these contrasting patterns make it possible to reconstruct ancestral genome organization and identify the evolutionary processes that produced present‐day chromosomes.

Ancestral linkage groups provide a framework for tracing chromosome fusions, fissions, and fusion‐with‐mixing events over hundreds of millions of years (Simakov et al. 2020, 2022). Because some of these rearrangements are unlikely to arise independently in the same configuration, they can also serve as rare genomic characters for resolving difficult phylogenetic relationships. Their application to early animal evolution and spiralian phylogeny demonstrates that chromosome structure can provide evidence complementary to conventional analyses based on sequence alignments (Lewin, Liao, Chen, et al. 2025; Lewin, Sakagami, Kao, et al. 2025; Schultz et al. 2023).

The next challenge is to move from reconstructing rearrangement histories to explaining why chromosome architecture remains stable in some lineages but changes rapidly in others. Denser taxonomic and population sampling will be required to determine when rearrangements occurred and whether they accumulated gradually or during short episodes of genome restructuring. Integration with information on transposable elements, centromeres, whole‐genome duplication, chromatin organization, and gene regulation will also be necessary to identify the mechanisms and biological consequences of chromosome change. In this way, chromosome‐scale comparisons can extend observations from individual species into broader models of genome evolution, phylogenetic history, and the constraints that shape chromosome architecture (Simakov and Wagner 2025).

### 4.4 Toward Experimental Chromosome‐Scale Genomics

Building on the comparative and glocal frameworks described above, the increasing accessibility of chromosome‐level genome assemblies, together with advances in single‐cell genomics and artificial intelligence tools such as genome language models (Consens et al. 2025; Merchant et al. 2025), is enabling local gene functions and cell‐type‐specific regulatory states to be interpreted within the context of global chromosome architecture. This integration provides not only new perspectives on genome function and evolution but also experimentally testable hypotheses concerning the regulation and organization of extensive genomic regions. Chromosome‐level genome assemblies can therefore serve not only as reference coordinates for interpreting genome function and evolution but also as design templates for experimentally testing hypotheses at the scale of large genomic regions.

Conventional genome editing generally targets individual genes or relatively short intervals, whereas chromosome‐level analyses increasingly reveal structural variants, gene clusters, repetitive regions, centromeres, and long‐range regulatory landscapes extending over hundreds of kilobases or megabases. Telomere‐to‐telomere assemblies have further resolved structurally complex regions that were inaccessible in earlier reference genomes, creating opportunities to move from reading chromosome sequences toward reconstructing and manipulating chromosome‐scale structures (Altemose et al. 2022; Nurk et al. 2022).

Large genomic regions can be generated through chemical synthesis or isolated from existing genomes using long‐fragment cloning methods, such as transformation‐associated recombination cloning in yeast (Kouprina and Larionov 2016). Human and mouse artificial chromosomes provide additional platforms for maintaining megabase‐sized genomic regions as independently segregating elements with functional centromeres and telomeres (Kouprina et al. 2013). These artificial chromosomes can be generated through bottom‐up approaches, in which centromeric DNA is assembled de novo, or top‐down approaches, in which native chromosomes are engineered while retaining their endogenous centromeres. Recent advances have enabled the generation of structurally defined human artificial chromosomes, while CRISPR/Cas9‐based approaches have expanded chromosome engineering to include chromosome truncation, reciprocal translocation, and megabase‐scale deletion (Egawa et al. 2025; Gambogi et al. 2024; Miyamoto et al. 2024; Uno et al. 2017). Engineered chromosomes can also be transferred between cells through methods such as microcell‐mediated chromosome transfer, facilitating the establishment of cellular and animal models carrying large genomic regions (Kazuki et al. 2022; Uno et al. 2024).

Although chromosome engineering remains technically demanding and has so far been developed most extensively in mammalian cells and animal models, it represents a promising future extension of chromosome‐level genomics. The case studies discussed in this review raise hypotheses concerning structural variation, transposable element expansion, long‐range gene regulation, chromosome rearrangement, and genome dosage. By enabling large genomic regions to be deleted, transferred, reconstructed, or redesigned, chromosome engineering may provide a route to test these hypotheses experimentally rather than relying solely on comparative inference. Beyond its value for mechanistic studies, chromosome engineering may also contribute to clinical applications, including the development of therapeutic strategies for chromosomal aneuploidies such as Down syndrome (Hashizume et al. 2025). As genome assemblies and manipulation technologies become available across a broader range of species, experimental chromosome‐scale genomics may connect biodiversity‐driven genome discovery with functional validation at the level of entire loci and chromosomes (Figure 4).

### 4.5 Conclusion

Chromosome‐level genome assemblies are transforming biological research not simply because they provide more complete reference sequences, but because they reveal the organization of genomes at the scale at which structural variation, gene regulation, chromosome evolution, and genome dosage can be meaningfully interpreted. The case studies reviewed here demonstrate how chromosome‐resolved genomes reveal lineage‐specific architecture, connect local molecular changes with phenotypic innovation and plasticity, reconstruct deep evolutionary history, and resolve genetically complex and polyploid systems.

Future progress will depend on moving beyond isolated reference genomes toward comparative, pangenomic, population‐scale, and haplotype‐resolved resources. These resources must in turn be integrated with multi‐omics data and functional experiments (Figure 4). Within this framework, glocal biology provides an approach for connecting genes, regulatory elements, sequence variants, and cellular states with chromosome architecture, population diversity, organismal phenotypes, and evolutionary history. Chromosome‐level assemblies can thereby move from descriptive genomic resources to platforms for generating and experimentally testing mechanistic hypotheses about genome function and evolution.

## Author Contributions

**Tetsuo Kon:** conceptualization, writing – original draft, writing – review and editing, funding acquisition. **Mayuko Hamada:** conceptualization, writing – review and editing, writing – original draft, funding acquisition. **Kosuke Kataoka:** conceptualization, writing – review and editing, writing – original draft, funding acquisition. **Yuki Monden:** writing – original draft, writing – review and editing. **Johannes Nicolaus Wibisana:** writing – original draft, writing – review and editing. **Narumi Uno:** writing – review and editing, writing – original draft. **Kouhei Toga:** writing – original draft, writing – review and editing. **Yi‐Jyun Luo:** writing – review and editing, writing – original draft.

## Funding

This work was supported by the JSPS KAKENHI (Grant Numbers JP24K09559 to M.H., JP26K02062 to K.K. and JP23K05867 to N.U.), the JSPS Overseas Research Fellowships to T.K., and the Bio‐oriented Technology Research Advancement Institution (JPJ009237) to K.K.

## Conflicts of Interest

The authors declare no conflicts of interest.

## Data Availability

No new experimental data were generated in this study. The data underlying Figure 1 and Table 1 were retrieved from the NCBI Genome database.
